# A Review of Recent Advances in Ultrasound, Placed in the Context of Pain Diagnosis and Treatment

**DOI:** 10.1007/s11916-018-0711-7

**Published:** 2018-07-10

**Authors:** Michael S. Bobola, Lucas Chen, Chikodinaka K. Ezeokeke, Katy Kuznetsova, Annamarie C. Lahti, Weicheng Lou, Aleksey N. Myroniv, Nels W. Schimek, Madison L. Selby, Pierre D. Mourad

**Affiliations:** 1 Department of Neurological Surgery, University of Washington, Seattle, WA USA; 2 Applied Physics Laboratory, University of Washington, Seattle, WA USA; 3 Division of Engineering and Mathematics, University of Washington, Bothell, WA USA

**Keywords:** Ultrasound, Peripheral nerve stimulation, Pain diagnosis, Pain, Pain treatment

## Abstract

Ultrasound plays a significant role in the diagnosis and treatment of pain, with significant literature reaching back many years, especially with regard to diagnostic ultrasound and its use for guiding needle-based delivery of drugs. Advances in ultrasound over at least the last decade have opened up new areas of inquiry and potential clinical efficacy in the context of pain diagnosis and treatment. Here we offer an overview of the recent literature associated with ultrasound and pain in order to highlight some promising frontiers at the intersection of these two subjects. We focus first on peripheral application of ultrasound, for which there is a relatively rich, though still young, literature. We then move to central application of ultrasound, for which there is little literature but much promise.

## Ultrasound for Peripheral Nerve Stimulation

In the early 1970s, researchers used both focused and unfocused ultrasound to evoke tactile sensation in human subjects. Gavrilov and colleagues investigated the use of stimulatory ultrasound to induce tactile, thermal, and painful sensations in the human hand [1–4]. For example, in Gavrilov 1977a, the authors studied the sensations generated by ultrasound delivered to the skin or below the skin as a function of intensity and water temperature. The piezoceramic transducers delivered ultrasound at resonant frequencies of 0.48, 0.887, and 2.67 MHz, and maximum intensities of 1300, 8000, and 30,000 W/cm^2^. Both the subject’s hand and the ultrasound transducer were submerged in warm bath water at temperatures of 30, 35, or 40 °C. Each subject described the presence or absence of sensation during ultrasound application to either their skin or the deep tissues for a duration of 1 ms, followed by 10 and 100 ms. Stimulation stopped immediately after the test subjects reported pain associated with ultrasound application. At lowest intensity values of ultrasound—the “threshold intensity”—test subjects felt a tactile sensation, described by them as a “local touch,” a “slightly sensed stroke,” or a “slight push.” Interestingly, the threshold intensity for induction of a tactile sensation increased as the frequency of ultrasound increased. Also, the threshold intensity increased with movement of the focal region within the skin layer from the fingers to forearm. At larger intensity values, the sensation felt by subjects involved modification of temperature sensing. Specifically, in a way that varied between test subjects, ultrasound applied directly to the skin induced sensations of warmth and cold, sensations that disappeared when ultrasound of the same intensity focused below the skin layer. As was the case for tactile sensations, threshold intensity values for ultrasound-induced temperature sensations increased as its delivery point moved from the finger to the forearm. Moreover, depending on water temperature, stimulation of a given sensitive spot generally created a cold sensation (at 30 °C) or warm sensation (at 40 °C). As a means of studying the biophysical processes that lay behind these observations, the authors calculated the particle velocity, sound pressure, displacement, and temperature of tissue induced by ultrasound and correlated those calculations with the observed threshold intensity values. All those physical parameters other than tissue displacement increased as a function of intensity. Only the displacement amplitude of tissue within the focal volume of ultrasound application at its threshold value was independent of frequency [1]. Therefore, biophysical process of a mechanical nature plays a significant role, at least, in the stimulation of peripheral tissue.

Others, motivated by Gavrilov and colleagues, have explored many of the concepts implicit or explicit in their work. For example, one of the main goals of the work led by Dalecki [5] was to test the hypothesis that the tactile sensation experienced through direct exposure of tissue to ultrasound is caused by that tissue’s exposure to the acoustic radiation force, itself related to the absorption by tissue of ultrasound. Experiments used an acoustically reflecting target, Corprene, set on the tissue site. This cork/rubber compound contained large amounts of trapped air so that it acted as an acoustic reflector. That reflection resulted in displacement of the Corprene, hence displacement of the underlying tissue. Importantly, the Corprene prevented the direct exposure of tissue to ultrasound yet produced local displacement of tissue without the induction of cavitation and of heat within tissue. Thresholds for tactile perception during ultrasound exposure of a portion of forearm away from bone with the Corprene target were compared to those of direct exposure of the forearm. They used ultrasound with a carrier frequency of 2.2 MHz and considered four pulse durations: 5,10, 50, and 100 ms. Across their protocol, the observed ultrasound threshold intensity values for perception of ultrasound did not depend upon the absence or presence of Corprene. This result supports the investigators’ hypothesis that ultrasound-induced tactile sensations arise due to the acoustic radiation force. In addition, Dickey et al. [6] also determined threshold values for perceived sensations, using pulses of ultrasound that lasted for 0.1 s, delivering actual or sham ultrasound at a carrier frequency of 2 MHz into the fingertip pads of healthy test subjects. They observed an increase in sensitivity to ultrasound delivery as a function of increased intensity values. Of note, this increase in sensitivity to ultrasound stimulation correlated inversely with the density of peripheral nerve terminals in the fingertip pads, itself determined through use of a two-point discrimination test, consistent with the hypothesis of Gavrilov (1984) [3].

The work reviewed above used intense focused ultrasound (iFU)—ultrasound with intensities above FDA limits for diagnostic ultrasound) which has dealt with stimulating healthy tissue, in vivo and in humans. Of note, Gavrilov (1984) [3] hypothesized that clinicians could 1 day use iFU application to distinguish between healthy and neuropathic tissue, based on the qualities of the sensations evoked by ultrasound stimulation, thereby providing a noninvasive method for focally locating neural abnormalities in patients. Motivated by that hypothesis, researchers have shown it possible to elicit diagnostically relevant responses using inflammatory rat models of pain [7–9], neuropathic rat models of pain [10, 11], and patients [12, 13]. In all these papers, the authors found a lower intensity of ultrasound was required to elicit a discernible sensation or withdrawal response after application of iFU to damaged tissue than when applied to control (healthy) tissue.

For example, McClintic et al. [8] set out to show that iFU stimulation could be used as a noninvasive, targeted test for identifying inflamed tissue. A 0.375-s pulse of 2-MHz ultrasound was applied in a randomized fashion to the hind paws of rats, one of which was inflamed; the other, not. They observed that after application of iFU, the inflamed paw withdrew at a lower threshold of ultrasound intensity 100% of the time, and that iFU threshold values were two times higher for normal paws than for inflamed paws, a statistically significant result. An acute safety study [9] found that 20 separate 0.1-s iFU applications at 1000 W/cm^2^ spaced 10 s apart produced no observable cell damage in rats in the subcutaneous area of ultrasound application, while 30 0.1-s iFU applications at 2000 W/cm^2^ did produce observable damage in four rats. Interestingly, Garcia et al. (2014) [7] used the same rat model of inflammation to observe a significant diurnal difference in iFU threshold values, with high specificity and sensitivity. Specifically, the thresholds for stimulation for both single pulse and multiple pulse protocols were significantly higher at night than during the day, consistent with the diurnal pattern of pain for rodents, and lending hope to the idea that iFU stimulation could track pain management through time.

Motivated by these in vivo results for inflammatory tissue, these same researchers have used image-guided iFU (ig-iFU) to study peripheral pain generators in humans with known inflammatory pain in their shoulder. Specifically, Gelhorn, Gillenwater, and Mourad (2015) [12] applied ig-iFU to candidate trouble spots in the shoulders of patients with rotary cuff tendinosis. They used a Sonosite ultrasound imaging device, coupled with a 2.0-MHz iFU transducer to deliver iFU in individual pulses of length 0.1 s with escalating intensity values until either the test subject gave definitive reports of sensation induction or the iFU device reached its maximum intensity value. These researchers identified the iFU intensity threshold values of sensation induction in rotary cuff tendons along with several other sites in each group of participants, with values significantly less than that observed for control subjects. It was determined that while neither the healthy volunteers nor osteoarthritis patients reported any sensation upon application of iFU, patients with rotary cuff tendinosis did report reliable sensation induction in their rotary cuff tendon and surrounding tissue at a spatial and temporal average intensity (*I*_SATA_) value of 680 ± 281 W/cm^2^.

Regarding neuropathic pain, Tych et al. (2013) [10] and McClintic et al. (2013) [11] sought to demonstrate that iFU could distinguish between diffuse neuropathic tissue and healthy tissue. Tych et al. (2013) used an ultrasound pulse of 1.15-MHz frequency, applied for 0.2 s into the hind paws of rats, one distal to pSNL (partial sciatic nerve ligation), the other left alone. Then, the rats were observed for instantaneous paw withdrawal. If there was no withdrawal, the intensity of the ultrasound was increased in 30% increments starting at 50 W/cm^2^ until two consecutive single paw withdrawals were observed, defining this intensity as the iFU threshold. Tych et al. (2013) observed in pSNL rats that the rat withdrew its ipsilateral 98% of the time in 59 trials without withdrawing its contralateral paw at the same iFU intensity value. An average intensity and dose of 176 ± 56 W/cm^2^ and 37.4 ± 11 (W/cm^2^)*s was required to see a response in pSNL. The intensity and dose of ultrasound required to elicit a response in the sham surgery rats was 217 ± 25 W/cm^2^ and 43.4 ± 5 (W/cm^2^)*s, respectively, while in the control rats, the ultrasound device could not elicit a response even at its maximum intensity and dose of 283 W/cm^2^ and 56.6 (W/cm^2^)*s, respectively. This study demonstrates that diffuse neuropathic tissue is more sensitive to iFU compared to healthy tissue.

McClintic et al. (2013) [11] sought to extend this work to focal and subcutaneous neuropathic tissue, specifically a neuroma. The authors applied a single, 2-MHz ultrasound pulse that lasted for 0.1 s to the neuroma while the rat was under light anesthesia. (They located the neuroma through anatomical markers and verified its sensitivity through use of von Frey hairs.) For control tissue, they stimulated an area of the rat’s leg 1 cm away from the neuroma towards the body. Starting at low intensity values, McClintic increased the iFU intensity in 10–30% increments targeting the neuroma until they observed three reliable flicks of its ipsilateral paw. After this observation, the authors applied the same intensity of iFU to the control area to see if it would elicit a response. In 21 out of 25 tests, the iFU elicited a response after application to the neuroma but not after its application to the control area. The results of this study agree with the results found by Tych et al. (2013) that neuropathic tissue is more sensitive than healthy tissue to iFU stimulation. These results further support the hypothesis that iFU stimulation can help differentiate painful tissue (here, subcutaneous neuropathic tissue) from normal tissue.

Motivated by McClintic et al. (2013) [11], Mourad et al. (2017) [13] used this same concept of ig-iFU stimulation to assay in a preliminary way the peripheral pain generators within the residual limbs of amputee patients. The researchers had access to five 2-MHz image-guided intense focused ultrasound transducers, each with a different depth of focus (specifically, 0.4, 1.3, 2.45, 2.75, and 3.0 cm) to target the transected nerve within the residual limbs of both standard amputee patients and those who had undergone a targeted nerve innervation (TNI) procedure. They applied 0.1 s of iFU stimulation to both the severed nerve endings and the immediately proximal section of the same nerve. When time permitted, they also applied iFU to the corresponding contralateral and intact nerve. They increased the applied intensity from 16 W/cm^2^ until a reliable intensity threshold for sensation induction was found, or else stopped at 1032 W/cm^2^, the maximum value of intensity achieved by the device. One or two neuromas were identified using ultrasound imaging for each of the four TNI patients. For three out of four TNI patients, they found an iFU threshold stimulation value below the maximum value produced by the transducer. For all three of those successful stimulation cases, the proximal nerve had the same iFU intensity value as for the neuroma itself. For the standard amputation group, neuromas were identified in three of the seven patients. For two of these three patients, the intensity threshold was the same in the neuroma and proximal nerve, while for the third patient, iFU applied to the proximal nerve did not generate a sensation, while for the distal neuroma, it did. For the remaining four out of seven standard amputees, iFU applied to the transected nerve end also generated a discernable sensation. Of particular interest, most successful iFU stimulation tests produced phantom limb sensations; also, for only one of the 11 test subjects could the authors elicit a discernable sensation with iFU applied to the contralateral and intact nerve, and then not in a reproducible fashion. Finally, the iFU threshold values trended inversely but were not statistically significant for phantom limb pain and pain associated with the participants’ residual limb in the standard amputee patients while both had a statistical inverse correlation in the TNI group.

Wright and colleagues [14] conducted a study that tested the ability of rapidly repeated application of focused ultrasound to induce temporal summation within skin, muscles, and joints. The stimulations occurred in single or sets of four pulses applied across a range of application frequency and duration with a center frequency of 1.66 MHz. They found a lower threshold for observable sensation induction when they rapidly applied multiple pulses than when they applied a single pulse, consistent with the idea that ultrasound could induce temporal summation. Expanding on this, McClintic and colleagues [8] used a rat model of chronic inflammation to compare the threshold for immediate paw withdrawal through a single burst of focused ultrasound and a series of rapidly applied bursts. They used a transducer with a center frequency of 2 MHz and a range of *I*_SATA_ intensity values between 100 and 1622 W/cm^2^ and found that five rapidly applied 75-ms iFU pulses spaced 75 ms apart produced withdrawal of the inflamed rat paw at lower iFU intensity values than a single 75-ms burst, consistent with the presence of temporal summation in this chronic pain model and the results of Wright and colleagues. However, the total acoustic dose and predicted heat increase were the same in the single burst and multiple burst protocols which produced paw withdrawal. This suggests that while temporal summation may allow for sensation induction at a lower iFU intensity in the multiple pulse condition through rapid pushing of this sensitive tissue, a temperature increase alone or in addition may have generated paw withdrawal behavior in these animals (Table 1).

**Table 1:** Summaries of representative articles in section “Ultrasound for Peripheral Nerve Stimulation”

Article	Model	US parameters	Result/conclusion
Tych et al. (2013) [10]	In vivo partial sciatic nerve ligation (pSNL) in Sprague-Dawley rats	1.15-MHz pulses 0.2 s Responses at: pSNL: 176 ± 56 W/cm^2^ *I*_SATA_ sham: 217 ± 25 W/cm^2^ *I*_SATA_ normal: greater than 283 W/cm^2^ *I*_SATA_	Neuropathic tissue is more sensitive to stimulation by intense focused ultrasound (iFU) than control tissue.
McClintic et al. (2013) [11]	In vivo neuroma in paw of Sprague-Dawley rats	2-MHz pulses 0.1 s Response at: Mechanical: 5.7 ± 2.2 g iFU: 343 ± 77 W/cm^2^	Successful stimulation of the neuroma by intense focused ultrasound required co-localization of the neuroma and intense focused ultrasound
Wright et al. (2002) [14]	Distal interphalangeal joint of index finger of human	Experiment 1 4 pulses at 2 Hz 25 ms 50 ms 75 ms 100 ms Experiment 2 0.5 Hz, 1 Hz, 2 Hz, 3 Hz, 4 Hz, 5 Hz Constant pulse of 50 ms	Experiment 1: “...a progressive decrease in pain thresholds was found with increased stimulus duration” Experiment 2: “Analysis of variance showed a significant interaction between tissue stimulated and pulse-train frequency.”

## Ultrasound Alone for Anesthesia and Analgesia

As noted in the introduction, there exists a rich history of the use of diagnostic ultrasound imaging to facilitate delivery of drugs that temporarily block the function of peripheral nerves in order to generate regional anesthesia. Interestingly, evidence exists pointing to the possibility that more intense ultrasound than that capable of producing a sensation, applied directly to a peripheral nerve, can transiently and safely reduce that nerve’s function. Because ultrasound is noninvasive, utilizing focused ultrasound to reversibly block nerve conduction for analgesia and anesthesia therefore has considerable appeal.

Colucci et al. 2009 [15] applied ultrasound to the sciatic nerve of bullfrogs. They tried two different ultrasound frequencies (0.661 and 1.986 MHz) and two different pulse durations (1 and 10 ms) and two different application rates (10 and 20 times per second), for 30 s in duration. Ultrasound stimulation significantly reduced up to 60% of the nerves’ action potential, which returned to baseline several minutes after ultrasound application. A thermacouple placed inside of the nerve recorded focal heat generated by the ultrasound, with that heat increase correlated with the observed action potential reduction. This work therefore not only demonstrated transient decrease in nerve function after application of focused ultrasound but also pointed towards at least one mechanism by which ultrasound created this effect.

Hong et al. 1991 [16] showed that focused ultrasound could reversibly block nerve conduction in humans. They applied, transcutaneously, 1-min physio-therapeutic ultrasound at a frequency of 2 MHz to the peroneal nerve of healthy test subjects at intensities of 0.5, 1.0, and 1.5 W/cm^2^. This stimulation produced a significant reduction in compound muscle action potential (CMAP), with a 41.4% decrease at 1.0 W/cm^2^and 44% decrease at 1.5 W/cm^2^, but not at 0.5 W/cm^2^. Normal CMAP production returned to baseline 5 min after ultrasound stimulation.

Going beyond *transient* reduction of nerve function, Foley and colleagues demonstrated that much higher intensity ultrasound applied to a major nerve could stop nerve function through induction of distal axon degeneration. Specifically, Foley et al. [17] monitored the motor function of the hind limbs of twelve rabbits following application of high-intensity focused ultrasound, intra-operatively, to the rabbit’s sciatic nerves. (They assayed motor function intra-operatively as well, through placement of a stimulating electrode proximal to the point of high-intensity focused ultrasound (HIFU) application and observation of paw movement in response to electrical stimulation.) They used HIFU with a spatial and temporal average intensity of 1930 W/cm^2^ delivered at 3.2 MHz, in 5-s intervals, until electrical stimulation could not induce a motor response. They observed a lack of motor response (assayed intra-operatively as above) for up to 14 days after HIFU application, consistent with associated histological examination, which showed distal axon degeneration (Table 2).

**Table 2:** Summaries of representative articles in section “Ultrasound Alone for Anesthesia and Analgesia”

Article	Model	US Parameters	Result/conclusion
Colucci et al. (2009) [15]	In vivo, bullfrog, sciatic nerve	0.661 MHz 1.986 MHz 1 ms, 10 ms for 10 and 20 pulses per second, for 30 s	“Thermal mechanism of focused ultrasound can be used to block nerve conduction, either temporarily or permanently.”
Hong et al. (1991) [16]	In vivo, human, peroneal nerve	At 0.5 W/cm^2^, 1.0 W/cm^2^, 1.5 W/cm^2^	“Ultrasonic therapy with therapeutic dosage may cause a reversible conduction block on patients with painful polyneuropathy. “
Foley et al. (2006) [17]	In vivo, rabbit, sciatic nerve	1930 W/cm^2^ 3.2 MHz 5-s intervals	Conduction nerve block of all 12 sciatic nerves was achieved with average HIFU treatment time of 10.5 ± 4.9 s (mean ± SD).

## High-Intensity Focused Ultrasound to Treat Peripheral Sources of Pain

Though ultrasound, as a means of imaging, can guide RF ablation [18], ultrasound of a different sort—HIFU—of sufficient intensity can permanently destroy tissue. Here we first review MRI-guided HIFU systems, known as MRgHIFU, that represent a highly precise means of delivering HIFU. We then move on to their peripheral and central applications to pain treatment.

### MRgHIFU Systems for Delivering High-Intensity Focused Ultrasound

The first applications of MRI-guided high-intensity focused ultrasound began in the 1990s, with feasibility studies done on tissues to assess the thermal effects of focused ultrasound for the use of minimally invasive surgery.

Important early work showed the usefulness of MRI guidance for HIFU. In 1992, Jolesz and Hynynen al. [19] treated an acoustic silicone phantom gel and a bovine muscle with high-intensity focused ultrasound using MR imaging guidance. The experiments were done with a 1.1- and a 1.5-MHz transducer. With the 1.1-MHz transducer, the real-time MR imaging was able to show physical changes in the phantom gel and the bovine muscle at the site of the focus, as well as provide a temperature reading throughout the procedure. The procedures were done at various power levels and real-time MR imaging allowed for observation of HIFU-induced temperature increases from 30 to 60 °C. The study found reversible effects of HIFU with temperatures below 60 °C, with non-reversible effects above that temperature.

In 1993, Hynynen et al [20] also studied the feasibility of HIFU application under MRI guidance, as well as the feasibility of detecting tissue necrosis induced by HIFU with MR imaging, all in real time. For their experiments, Hynynen et al. used six, co-focused 1.1-MHz ultrasound transducers, each made of MRI-compatible materials, and a GE Signa 1.5T MR imaging system for the experiments, with the transducers placed within the MR bore. The experiments sonicated greyhounds’ thigh muscle while monitoring the tissue temperature rise and structural changes via MR imaging. The study found that sonications of 20 s or longer produced visible (by MR) lesions in tissue. Lesions were immediately detected and the magnitude of the change (of lesions) correlated with the duration of the sonication. Postmortem analysis of the tissue found that the size and shape of lesions correlated with the MR images. The study showed that MR systems can be used to monitor HIFU therapy as well as give real-time feedback on the dimension and location of targeted volumes.

#### Advantages of MRI Guidance Over Ultrasound Guidance

MR imaging is more advantageous than ultrasound imaging [21] for guidance of therapy using HIFU because MRI provides more information. MR imaging provides high-resolution anatomical imaging as well as thermal mapping with uninterrupted feedback during therapy [20]. Thermal mapping of the tissue is done directly from MR images, providing information about the thermal diffusion within the tissue, which ultimately defines the length of the HIFU therapy [20, 22]. Unlike other imaging methods, MR imaging provides excellent resolution of soft tissues, such as brain, joints, and spine. MRI guidance also allows for imaging from different planes (axial, sagittal, coronal, and oblique) without repositioning the subject. This is very important and beneficial during therapy for optimal targeting and monitoring of surrounding tissues. In instances of transcranial focused ultrasound surgery (FUS) therapy, MR imaging provides real-time feedback of location of focus of the transducer within the brain.

This is all in contrast to ultrasound imaging, with its lower resolution, significant operator dependency, and limited view of the tissue of interest. Moreover, diagnostic ultrasound does not currently have the ability to effectively monitor temperature [21].

#### Systems Available from Manufacturers

Currently, there are four MRIgHIFU systems available: the Sonalleve, the TULSA-PRO, the ExAblate O.R., and the ExAblate Neuro. The first three target the periphery while the fourth specializes in the brain.

The Sonalleve system was developed by Phillips to treat uterine fibroids and palliative pain of bone metastases. Philips sold the system to Profound Medical in June, 2017. The Sonalleve system includes an MR system with HIFU transducer built for noninvasive ablation. The HIFU transducer system includes a water cooling system to keep the patient’s skin temperature constant, and the system is embedded into the MR table. The MR system provides 3D images for planning and real-time feedback of tissue temperature during HIFU therapy [23].

Profound Medical has an additional MRIgFU system, TULSA-PRO, for the treatment of prostate cancer and ablation of prostate tumors. The system incorporates a robotic therapeutic ultrasound transducer that provides ablation of prostate tumors. The MR system provides real-time feedback of temperature of the target volume as well as the surrounding tissues. The system also provides tissue cooling through the rectum and urethra for the protection of tissues surrounding the volume target [24].

Another MRIgHIFU system that was developed for treatment of uterine fibrosis and palliative pain of bone metastases is the ExAblate O.R., created through a collaboration between GE and Insightec. This system includes an MR system and a table-embedded HIFU transducer for ablation therapy. Similar to other systems, the MR system provides 3D images for therapy planning and tissue temperature monitoring during therapy.

### Ablation of Peripheral Tissue by Ultrasound to Ameliorate Bone Pain Due to Cancer

Several researchers have explored tissue ablation with HIFU to treat pain, especially bone pain [25]. In all cases, they used MRI-guided high-intensity focused ultrasound. Osteoid osteoma, for example, is a type of bone cancer that produces significant pain often along the cortical long bones. Current conservative treatments include aspirin, also known as acetylsalicylic acid (ASA) or nonsteroidal anti-inflammatory drugs (NSAIDs). Surgical interventions include surgical en bloc resection or curettage. The success of surgery for reducing or removing the cancer pain (88–100%) comes with the price of increased complication rates (35%) relative to conservative treatment [26]. HIFU introduces a noninvasive alternative to surgical treatment of this pain, one that may spare adjacent anatomical structures [26].

Hurwitz et al. [27] tested the ability of HIFU ablation of painful, generally metastasized cancer to reduce that pain. Here, patients with metastatic growths emanating from cancers such as breast, kidney, lung, and prostate were evaluated prior to treatment using MRI to determine the size and locations of the area designated for HIFU treatment. Pain levels were also recorded using a numerical scale to quantify pain experienced by the patient [28]. After establishing those baseline values, patients underwent 2–4-h (including 83 ± 43 min for sonication) MRI-guided focused ultrasound treatment and were evaluated for pain in the treated tissue using the Numeric Rating Scale (NRS) and the Brief Pain Inventory (BPI-QoL). The results of their study conveyed a statistical significance (*p* < 0.05) in both measures when patients receiving the ablation treatment were compared to the sham cohort. Although there were 51 reported adverse events in the cohort (*n* = 112) that received focused ultrasound, more than half of them were resolved on the day they arose.

Napoli et al. (2017) [29] found similar results in a clinical trial: a decrease in pain after application of HIFU therapy to painful but benign bone tumors. They studied 29 patients who were diagnosed with osteoid osteoma and treated with MRgFUS therapy. Twenty-seven of the 29 patients reported an absence of pain without consuming any analgesics following the treatment. Their recorded pain score on the visual analog scale (VAS) significantly decreased from a 7.9 baseline to a score of 0.7 up to 24 months after treatment [28]. Encouragingly, they did not observe any complications associated with the treatment. These results support using HIFU as a precise and minimally invasive method to ablate, hence treat pain caused by bone cancer. Similarly, Li et al. (2010) [30] conducted a study on patients with malignant bone tumors. They observed HIFU therapy to successfully ablate the tumor such that the patients experienced a reduction in their pain. In this study, 25 patients with malignant bone tumors received HIFU treatment. Of the 25 patients, 24 of them experienced pain from their bone tumors before treatment. In order to measure the pain experienced, Li et al. (2010) used a verbal scale from 0 to 3 to rate pain: 0 indicating no pain, 1 indicating mild pain, 2 indicating moderate pain, and 3 indicating severe pain. The pain scores in the patients decreased from 1.84 ± 0.85 before HIFU therapy to 0.12 ± 0.33 after HIFU therapy. In the 24 patients experiencing pain, 21 of them (87.5%) were completely relieved of pain.

Yarmolenko PS et al. [31] and Sharma et al. [32] also explored the feasibility and safety of using MRI-guided HIFU to ablate osteoid osteoma. Nine patients under the age of 25, resistant to medical treatment, with lesions targetable with MR-HIFU were selected to participate in this study. Five days prior to treatment, both patient groups of MR-HIFU had evaluations of VAS recorded. For MR-HIFU patients, they were put under general anesthesia with a Sonalleve V2 HIFU system paired with an Achieva 1.5T MR scanner. Temperature scans of low-power sonications sufficient to induce a temporary rise in tissue temperature without causing ablation were applied prior to actual HIFU ablation to guide the HIFU treatment. Procedure time lasted, on average, 128 min with varying acoustic power and sonication duration dependent upon variables such as overlying bone thickness and osteoid osteoma characteristic.

The patients were interviewed several times (at 1, 7, 14, and 28 days post-treatment) to determine their VAS pain score and pain medication use. Patient median VAS score decreased from an average value of 6 down to 0 (*P* = 0.0002). Moreover, after treatment, eight out of nine patients stopped using NSAID. Finally, the patients experienced statistically significant increase in their sleep quality after HIFU treatment.

Taken together, these results and others’ [33–35] point to the ability of high-intensity focused ultrasound, under MRI guidance, to ablate boney lesions in a way that reduced their associated pain (Table 3).

**Table 3:** Summaries of representative articles in sections “MRgHIFU Systems for Delivering High-Intensity Focused Ultrasound” and “Ablation of Peripheral Tissue by Ultrasound to Ameliorate Bone Pain Due To Cancer”

Article	Model	US parameters	Result/conclusion
Liberman et al. 2009 [33]	In vivo, bone metastases	- Avg. time: 66 min (range 22–162 min) - Avg. sonications: 17.3 (range 8–32)	- VAS score reduction - Edema at target area - No lasting damage, some calcification of target area
Hurwitz et al. 2016 [27]	In vivo, bone metastases	- Avg. sonication time: 83 ± 43 min - Max 65–85 °C	- NRS score reduction, *p* < 0.001
Napoli et al. 2017 [29]	In vivo, human Osteoid osteomas	- 4 ± 1.8 sonications	- VAS score reduction (*p* = 0.001) - 27/29 patients had pain absence and no intake of NSAIDs
Li et a. 2010 [30]	In vivo, human Osteosarcoma, malignant fibrous histiocytoma	- 70 to 169 W/cm^2^ - Scanning speed = 1–3 mm/s - Avg. sessions = 2.29 h	- Pain reduction of *p* < 0.05 - PET-CT revealed no abnormal radioactivity concentration in tumor areas.

## (Towards) Use of Focused Ultrasound Applied to the *Brain* to Treat Pain

The skull represents a considerable barrier to ultrasound propagation into the brain, the major problem for those seeking to use focused ultrasound to treat portions of the brain associated with the experience of pain. Ultrasound not only attenuates in amplitude through absorption by the bone but also reflects and scatters the incident ultrasound as well as converts it from pressure to shear waves. These factors, as well as the variable thickness of the skull throughout its circumference, including inter-patient variability, while in play for diagnostic ultrasound, pose a quite significant problem for intense ultrasound, which puts the skull at risk for significant heating [36]. Current approaches alter the phase of the incident ultrasound based upon either CT scan-based mathematical modeling of ultrasound propagation or measurements of brain-tissue displacement caused by low levels of the acoustic radiation force [37]. In this way, intentional and designed initial defocusing of the ultrasound external to the skull yields a focused beam of ultrasound within the brain.

As mentioned above, GE and Insight created the ExAblate O.R., with its table-embedded HIFU transducer for ablation therapy. To treat brain, they modified this system to create the ExAblate Neuro, an MRIgHIFU system built to deliver HIFU across the skull. The system includes a novel HIFU system that incorporates one of the ultrasound focusing paradigms just discussed. It also has a helmet to help cool the patient’s head. Specifically, it includes a water cooling system that keeps the patient’s skin and skull cool, while the transducer’s 650 kHz, 1024-element phased array provides precise targeting and treatment of the selected volume [38].

Thanks to the ability of MR to detect small changes in temperature, users of the ExAblate Neuro can refine the position of the ultrasound focus via measurement of the location of a sub-ablation thermal spot induced by HIFU. Once the clinicians have confirmation of the desired location of the HIFU focus, they then increase the acoustic power in a stepwise fashion until the tissue reaches the therapeutic target temperature (52–59 °C). To ensure the safety of patients, sonication temperatures are monitored throughout the procedure with MR thermometry while the patients are fully responsive, awake, and questioned repeatedly to avoid adverse effects.

### Ablation of the *Brain* to Treat Pain

The ExAblate Neuro system has had its most extensive tests applied to the treatment of essential tremor due to Parkinson’s disease (PD), with one application to pain treatment. Since MRgHIFU treatment of essential tremor (ET) and pain each require ablation of brain tissue, we include essential tremor here to provide to the readers a sense of the range of HIFU parameters necessary to ablate brain tissue associated with pain.

Chang et al. [39] studied the efficacy of using unilateral magnetic resonance-guided ultrasound thalamotomy for essential tremor treatment. Following treatment, patients were assessed on tremor severity and functional impairment using a critical scale for tremor (CRST [40]). Follow-ups occurred at 1 week, 1 month, 3 months, and 6 months after treatment. Eight out of the 11 patients that could be considered for analysis showed significant improvement in parts A, B, and C of the CRST.

Magara et al. [41] demonstrated that the use of MRgFUS can provide similar improvements to these patients compared to radiofrequency pallidothalamic tractotomy. Thirteen patients (range 37 to 82 years) with therapy-resistant PD were approved for MRgFUS treatment, divided into two cohorts. Group 1 (patients 1–4) received a single application of peak temperature while group 2 (patients 5–13) received applications of peak temperature four to five times. After treatments, follow-ups were held at 2 days and 3 months. At the 3-month follow-up post-treatment, group 1 showed a mean UPDRS (unified Parkinson’s disease rating scale [42]) reduction of 7.6% while group 2 showed a mean reduction of 60.9%.

Chang et al. [39] observed comparable results in a prospective study of eleven patients’ essential tremor, 8/11 of which received sufficient HIFU to ablate tissue, and all experiencing immediate reduction in tremor-related symptoms out to 6 months—the end of the study period. Interestingly and in contrast to some other studies, the MRI manifestation of the HIFU lesions disappeared 3 months after HIFU application. Chazen et al. [43] observed similar clinical results for MRgFUS treatment of essential tremors as well as demonstrated the ability of diffusion tensor imaging to target ablation sites for MRgFUS. Post-operative imaging of their four patients showed a lesion at the desired location and physician evaluation demonstrated a significant improvement of symptoms.

For patients with chronic and therapy-resistant neuropathic pain, there are few options to reduce their pain. In response to this, a team in Switzerland [44] recruited nine patients (aged 45 to 75) for a selective central lateral thalamotomy. Before treatment with MRgHIFU began, they confirmed the location of the HIFU focus with low sonications with 10 to 20-s durations applied to temperatures of 39 to 42 °C, below the threshold for ablation, but are visible on the MR thermometry to confirm an accurate focus point. After achieving confirmation, the authors gradually increased the HIFU power in order to achieve peak temperature of 53–60 °C with continuous wave sonication at individual durations of 10 to 20 s. During the procedure, patients experienced a variety of effects such as temporary pain relief, vestibular feelings, and dysesthesias. The treatment created lesions of 3 to 5 mm in length, with pain relief ranged from 30 to 100% relative to their baseline scores. In addition, they did not observe any adverse effects. These preliminary findings indicate that MRgHIFU could be a safe and reliably precise noninvasive option for neurosurgery interventions [44].

Three years later, Jeanmonod et al. [45] achieved similar results with central lateral thalamotomy, which they performed on 12 patients with chronic therapy-resistant neuropathic pain. Prior to treatment, they applied several low-power sonications of 10 to 20 s, achieving MRI-detected temperatures of 39 to 42 °C, thereby confirming the location of the focus relative to the anatomical target. After this step, they began therapy, continuing until they observed a temperature range of 51–64 °C at the HIFU focus. The mean VAS score of the preoperative patients was 59.5/100 with “significant” relief in pain (mean = 55%) after the procedure. Post-operative follow-ups occurred with patients at the end of the procedure, 2 days, 3 months, and 1 year. In all instances of follow-up, the VAS score was significantly decreased relative to baseline, with a mean score of 35.3/100 on year post-operation. In addition, five out of eight patients from the study did not use drugs to combat pain at the 1-year mark, while all did before treatment (Table 4).

**Table 4:** Summaries of representative articles in section “Ablation of the Brain to Treat Pain”

Article	Model	US parameters	Result/conclusion
Magara et al. 2012 [41]	In vivo, human Pallidothalamic tract	710 kHz Mean application time: 13 s (range 10–21) Peak temp: 52–59 °C	Group 1: avg. lesion of 83 mm^3^ with disappearance at 3 months Group 2: avg. lesion of 172 mm^3^ with maintained visualization at 3 months
Chazen et al. 2017 [43]	In vivo, human Thalamotomy	650 kHz mean application time: 10–20 s Peak temp: 55–62 °C	Reduction in contralateral intention tremor
Chang et al. 2014 [39]	In vivo, human Thalamotomy	650 kHz Mean application time: 10–20s Peak temp: 55–62 °C	Immediate and sustained improvements in tremors Lesion of thalamic nucleus
Martin et al. 2009 [44]	In vivo, human Central lateral thalamotomy (CLT)	650 kHz Mean application time: 10–20 s Peak temp: 53–60 °C	3–5-mm lesion in 48-h post-op MRI Long-term (~ 3.75 years) pain relief between 50 and 100% in 53% of patients
Jeanmonod et al. 2012 [45]	In vivo, human Central lateral thalamotomy (CLT)	650 kHz Mean application time: 10–20 s Peak temp: 51–64 °C	Lesions of 3–4 mm (d) Mean post-op VAS score reduction of 42.3 and 40.7% at 3 months and 1 year respectively

### Towards Modulating Brain Function with Ultrasound to Treat Pain

In this section, we discuss observations that show that ultrasound can activate or deactivate the brain in a temporary and non-destructive fashion. As surely this audience knows, pain is a personal experience that requires brain function. Therefore—and speaking speculatively at this point—deactivation of relevant brain regions by ultrasound, or activation of a portion of the brain that inhibits downstream brain function, could have direct application to reducing a person’s experience of pain. In anticipation of future studies that explore these possibilities, we review here recent observations of ultrasound’s temporary effect on brain function.

Ultrasound-facilitated modulation of brain function (UNMOD) has experienced a resurgence since the early work in the 1950s performed by the Fry brothers [46] who, for example, observed that ultrasound delivered to the visual cortex of anesthetized cats could temporary deactivate it for 30 min. Leading this resurgence, Tyler and colleagues [47] directly measured neuron activation by ultrasound through placement of an electrode within the hippocampus of a slice of mouse brain, itself within the field of largely unfocused ultrasound at delivered at 500 kHz. Next, through the use of transcranial pulsed ultrasound that encompassed the majority of mouse brain, they induced generally bilateral peripheral motor activity such as tail and paw flicks and whisker movements [48]. King et al. [49] and Ye, Brown, and Pauly [50] produced comparable behavioral results, including with a wider exploration of neuromodulatory frequency. Younan et al. [51] produced similar observations coupled with a detailed numerical study of the ultrasound patterns within rodent brain. Moore et al. [52] demonstrated that ultrasound-induced EEG signals have temporal structure consistent with known activity in pyramidal neurons, as shown by comparison with their observed optogenetic stimulation.

To further increase the anatomical specificity of UNMOD, Tufail and colleagues [48] used an ultrasound collimator to produce directly measured action potentials generated within one hemisphere of the intact mouse brain. Yoo et al. [53] used a pulsed, 690-kHz focused ultrasound protocol on anesthetized rabbits, showing via functional magnetic resonance imaging (fMRI), electromyography (EMG), and gross observation that ultrasound delivered to one side of the brain induced observable brain function on the contralateral side, with focal volumes smaller than a hemisphere of brain. For their second study [54], the authors used a 350-kHz focused ultrasound protocol to stimulate at least a cranial nerve associated with control of an eye of an anesthetized rat, with motion induced ipsilateral to the stimulation zone. Kim et al. [55] showed with PET imaging that, among several results, the focal volume of the brain activated by 500-kHz ultrasound measures much smaller (given by a contour defined by the 90% of the peak value of pressure in the focus) than the focal volume of ultrasound defined physically (given by a contour defined by 50% of the peak value of pressure). This same group demonstrated in a rat model significant reduction of visually evoked potentials after application of ultrasound with one set of parameters (focused ultrasound with a 350-kHz carrier frequency, pulse repetition frequency of 100 Hz, spatial-peak pulse-average acoustic intensity of 3 W/cm^2^), with a slight enhancement of visually evoked potentials after application of ultrasound with slightly greater intensity and dose. They were motivated by earlier work [53] that demonstrated, in rabbits, UNMOD’s ability to reduce brain activity generated by light exposure and monitored by functional magnetic resonance imaging.

Mehic et al. [56] further demonstrated increased anatomical specificity of ultrasound stimulation of rodent brain, including large variations in motor response caused by small, lateral displacements of the ultrasound target—on the order of a millimeter. Kamimura et al. [57] achieved similar results as well as observed transient eye movement induced by ultrasound. Moreover, through the use of vibroacoustography at high frequencies (2.25 MHz and 1.75 MHz), Mehic et al. [56] were able to introduce low-frequency ultrasound (500 kHz, like many studies quoted above) into a smaller volume of the brain than that amenable to typical low-frequency ultrasound transducers. Finally, Yu et al. (2016) [58] used sophisticated analysis of signals derived from dense EEG arrays to map the propagation of brain activity induced by transcranially delivered ultrasound, from its point of application to other portions of the brain.

Several groups have demonstrated successful UNMOD in larger animals—sheep and non-human primates (NHPs). For example, Lee et al. [59] applied transcranial focused ultrasound to sensorimotor and visual areas of the brains of sheep and measured EMG signals in the hind legs elicited by ultrasound application to the brain, as well as visually evoked potentials. Also, Tanter and colleagues [60] altered the saccade patterns of awake NHPs through use of transcranial delivery of focused ultrasound with a carrier frequency of 320 kHz simultaneously with measurements of neural activity [61].

Finally, three intrepid groups [62–64] have applied neuromodulatory ultrasound in a transcranial fashion to the somatosensory cortex of healthy test subjects. The Tyler group [62] observed significant attenuation of the amplitudes of somatosensory evoked potentials elicited concurrently by median nerve stimulation. They also observed increased performance on sensory discrimination tasks without affecting task attention or response bias [63] as well as modulation of brain dynamics [64]. Yoo and colleagues [63] also stimulated the somatosensory cortex, thereby including tactile sensations in the hands of volunteers.

Three teams of researchers have applied UNMOD with therapeutic intent as of this writing. Min et al. (2011) [65] first induced acute epileptic seizures in a rat model. They then applied ultrasound, transcranially, to these anesthetized animals, with the following parameters: a carrier frequency of 690 KHz, with 500-ms-long pulses applied 100 times per second with an acoustic intensity of 130 mW/cm^2^. EEG monitoring demonstrated reduced seizure activity as compared to untreated controls. In another approach to epilepsy treatment, Airan et al. [66] demonstrated the use of focused ultrasound to release neuromodulatory drugs from engineered nanoparticles that stopped chemically induced seizures in a rat model. After injection of the nanoparticles IV, they applied iFU with a carrier frequency of 1 MHz, in short, repeating bursts at a frequency of 0.5 Hz, for 2 min, to the brains of these anesthetized mice. As in Min et al. (2011) [65], they observed statistically significant decreases in total EEG power in mice experiencing seizures relative to control mice. Finally, and quite evocatively, Monti et al. (2016) [67] reported, in their letter to the editor, delivery of focused ultrasound to the thalamus of a patient who was in a comatose state for 19 days after TBI. The patient showed remarkable recovery from this state over a period of 3 days, with immediately observable clinical improvement. Their ultrasound had a 650-kHz carrier frequency, an estimated in situ intensity of 0.72 W/cm^2^, and pulse lengths of 0.5 ms delivered 100 times per second for 30 s, with a 30 s pause, repeated ten times. This has some similarity to the results of Yoo et al. (2011) [68] who applied UNMOD to the thalamus of anesthetized rats, thereby reducing the time required for them to evince voluntary movement as they recovered from ketamine/xylazine anesthesia (Table 5).

**Table 5:** Summaries of representative articles in section “Towards Modulating Brain Function with Ultrasound to Treat Pain”

Article	Model	US parameters	Result/conclusion
Kamimura et al 2016 [57]	In vivo Mice strain C57BL-6	1.9 MHz Pulse rep freq of 1 kHz 50% duty cycle (950 pulses) On 1 s, off 1 s, ten times	Muscle movement at 1.9 MHz Pupil dilation at 1.2 MPa Pupil dilation at > 1.8 MPa
Airan et al 2017 [66]	In vivo Fischer 344 rats	1 MHz 0.5-Hz bursts for 2 min	Decrease in EEG at 1.0 and 1.5 MPA
Tufail et al 2010 [48]	In vivo Anesthetized mice	Pulses between 80 and 225 acoustic cycles per pulse of 0.16–0.57 ms Pulse repetition frequencies between 1.2 and 3.0 kHzSpatial-peak temporal-average intensities (*I*_SPTA_) of 21–163 mW/cm^2^	Motor cortex activation; Tail twitches and EMG activity in the lumbo sacrocaudalis dorsalis lateralis muscle; EMG response in the contralateral triceps brachii muscle
King et al 2013 [49]	In vivo CBL-7 mice	500 kHz Bandwidth of 340 to 650 kHz Ultrasound intensities from 0.01 to 79.02 W/cm^2^ (0.03 to 1.11 MPa) 20 to 480 ms	Brain activation from ultrasound can occur for ultrasound frequencies between 250 and 500 kHz.
Mehic et al 2014 [56]	In vivo C57BL/6 mice	88 bursts of 500-kHz ultrasound Length 200 μs Pulse repetition frequency of 1.5 kHz in a 1-s interval *I*_SPTA_ = 5.25 W/cm^2^	Production of tail movement, unilateral and bilateral movement of legs and whiskers correlated with small O (1 mm) lateral movement of iFU focus.
Tyler et al. 2008 [47]	Hippocampus slice cultures of mice brain	Low-frequency US (0.44–0.67 MHz) Spatial-peak pulse-average intensity (*I*_SPPA_) = 2.9 W/cm^2^	Brain activation at 500 kHz via EEG.

## Summary and Conclusion

Here we have reviewed a range of literature that highlights the potential for ultrasound to address painful conditions beyond its current clinical use for image-guiding injections.

We began by reviewing the literature relevant to the use of ultrasound to stimulate tactile touch. This work was pioneered by Gavrilov [1–4], starting in 1974 and continuing for more than a decade. The work by Dalecki’s group [5] provided evidence to support Gavrilov’s hypothesis that the tactile sensation arises due to tissue’s exposure to the acoustic radiation force, the transfer of momentum from the sound field to tissue medium. We next reviewed research related to the use of ultrasound to diagnose tissue abnormalities, again as hypothesized by Gavrilov. These include identifying inflamed or neuropathic tissue in rodent models [7–9] and in humans [6, 12, 13].

In addition to this novel use of ultrasound for diagnostic purposes, more intense ultrasound can induce local anesthesia or analgesia affects. The nerve-blocking ability of ultrasound is associated with its ability to produce heat, as shown in Colucci et al. 2009 [15], in vivo. In humans, Hong et al. 1991 demonstrated temporary, ultrasound-induced reduction of peripheral nerve conduction.

A further increase in ultrasound intensity can ablate peripheral tissue, demonstrated to reduce bone pain associated with metastatic cancer. Hurwitz et al. 2014 [27] used MRI-guided HIFU to ablate the cancerous growths in a manner that reduced these patients’ pain. Similar results were observed by Napoli et al. (2017) [29], Sharma et al. 2017 [32], and Yarmolenko PS et al. 2018 [31] for osteoid osteoma.

Pain can also be treated using focused ultrasound applied transcranially to the brain via ablative procedures [44, 45], a little studied but promising approach. Finally, transcranial ultrasound with significantly reduced intensity relative to ablative ultrasound can non-destructively and transiently activate as well as suppress brain function, shown in a range of animals (rodents [47–50, 52, 55–58, 65, 66, 68–70], rabbits [53], sheep [59], non-human primates [60, 61]) and modulated brain function in people [62–64, 67]. Perhaps, 1 day, neuromodulatory ultrasound can ameliorate the patient’s pain, at least temporarily.

In short, recent advances in ultrasound biophysics have opened up new opportunities to ameliorate the patient’s pain, worthy of further study and trial.
